# Evidence for Widespread Exonic Small RNAs in the Glaucophyte Alga *Cyanophora paradoxa*

**DOI:** 10.1371/journal.pone.0067669

**Published:** 2013-07-03

**Authors:** Jeferson Gross, Sana Wajid, Dana C. Price, Ehud Zelzion, Junyi Li, Cheong Xin Chan, Debashish Bhattacharya

**Affiliations:** 1 Department of Ecology, Evolution, and Natural Resources and Institute of Marine and Coastal Science, Rutgers University, New Brunswick, New Jersey, United States of America; 2 The University of Queensland, Institute for Molecular Bioscience, and ARC Centre of Excellence in Bioinformatics, Brisbane, Australia; NIGMS, NIH, United States of America

## Abstract

RNAi (RNA interference) relies on the production of small RNAs (sRNAs) from double-stranded RNA and comprises a major pathway in eukaryotes to restrict the propagation of selfish genetic elements. Amplification of the initial RNAi signal by generation of multiple secondary sRNAs from a targeted mRNA is catalyzed by RNA-dependent RNA polymerases (RdRPs). This phenomenon is known as transitivity and is particularly important in plants to limit the spread of viruses. Here we describe, using a genome-wide approach, the distribution of sRNAs in the glaucophyte alga *Cyanophora paradoxa*. *C. paradoxa* is a member of the supergroup Plantae (also known as Archaeplastida) that includes red algae, green algae, and plants. The ancient (>1 billion years ago) split of glaucophytes within Plantae suggests that *C. paradoxa* may be a useful model to learn about the early evolution of RNAi in the supergroup that ultimately gave rise to plants. Using next-generation sequencing and bioinformatic analyses we find that sRNAs in *C. paradoxa* are preferentially associated with mRNAs, including a large number of transcripts that encode proteins arising from different functional categories. This pattern of exonic sRNAs appears to be a general trend that affects a large fraction of mRNAs in the cell. In several cases we observe that sRNAs have a bias for a specific strand of the mRNA, including many instances of antisense predominance. The genome of *C. paradoxa* encodes four sequences that are homologous to RdRPs in *Arabidopsis thaliana*. We discuss the possibility that exonic sRNAs in the glaucophyte may be secondarily derived from mRNAs by the action of RdRPs. If this hypothesis is confirmed, then transitivity may have had an ancient origin in Plantae.

## Introduction

Eukaryotic genomes are “parasitized” by self-replicating genetic entities such as viruses and transposable elements (TEs, comprising transposons and retrotransposons). To suppress the intragenomic spread of such selfish replicons, eukaryotes have evolved diverse defensive strategies, among the most important of which is RNAi [1], [2]. This system relies on the recognition of double-stranded RNA (dsRNA) species, found in intermediary replication forms of virus, hairpin structures, or complementary antisense transcripts associated with TEs [1], [3], [4]. Such dsRNAs are processed by Dicer-type RNase III proteins to produce sRNAs that are usually 20–23 nucleotides in length [5]. The resulting sRNAs are loaded into a RNA-induced silencing complex (RISC), formed by Argonaute-type proteins (AGO), that recognize viral RNAs or TE-derived transcripts based on the sRNA-to-target base pair complementarity [2]. The silencing is accomplished by diverse mechanisms such as inhibition of transcription (e.g., by DNA or histone methylation), cleavage of the mRNA, or by blocking transcript translation [4], [6], [7], [8]. The RNAi process, whereby sRNAs are formed from perfect dsRNAs and act *in cis* by pairing to their cognate producing transcripts is referred to as the small interfering RNA (siRNA) pathway [1]. MicroRNAs (miRNA) also represent a class of sRNAs widespread in eukaryotic genomes that probably evolved from the ancestral siRNA pathway [1], [9]. However miRNAs differ from siRNAs by being derived from self-complementary fold-back precursor RNAs in which a few mismatches are included. These sRNAs recognize their targets by near-perfect complementarity and act primarily *in trans* to silence endogenous host protein-encoding transcripts [7], [8], [9]. A further class of sRNA found in plants, nematodes, and in the fungus *Mucor circinelloides* is associated with the amplification of the RNAi signal that is mediated by RNA-dependent RNA polymerases (RdRPs) in a phenomenon referred to as transitivity [10], [11]. The process is initiated by primary siRNAs or miRNAs that in an AGO-dependent fashion target viral RNAs or specific transcripts in the cell (e.g., trans-acting siRNA [tasiRNA] transcripts in plants), triggering the recruitment of an RdRP to drive the synthesis of a complementary (antisense) strand to the targeted transcript [11], [12], [13], [14]. The resulting duplex is subsequently processed by Dicer homologs into diverse secondary siRNAs that usually cover the length of the original transcript in patterns of phased registers. Secondary siRNAs reinforce silencing of the primary targeted RNA or, in the case of tasiRNA in higher plants, modulate expression of protein-coding mRNAs in *trans* [11], [14]. Therefore, transitivity yields the spread and amplification of the RNAi pathway that results in a robust form of defense against viral RNAs.

Differentially expressed sRNAs in response to fluctuating environmental conditions are already known in some prokaryotes such as marine strains of the cyanobacterium *Synechococcus* [15]. In eukaryotes, RNAi is believed to be an ancient process that probably evolved before the basal split of the major supergroups [16], [17]. In some lineages, reductive evolution resulted in partial or complete loss of the RNAi pathway, whereas in many multicellular organisms, amplification of the molecular components involved in RNAi contributed to the evolution of novel and intricate regulatory functions [1], [11], [16], [17]. For example, *Arabidopsis thaliana* encodes 4 Dicers, 10 Argonautes, and 6 RdRP homologs, reflecting an elaborate RNAi system that operates in plants [2], [11]. These data raise an important question: was RNAi co-opted from its original role as a molecular immune system against selfish elements to ultimately foster increasingly complex regulatory networks in the cell? RNAi and transitivity are also found in the moss *Physcomitrella patens* and the green alga *Chlamydomonas reinhardtii* [18], [19], [20]. Other algae, such as *Phaeodactylum tricornutum*, *Thalassiosira pseudonana*, *Pyropia yezoensis*, *Ectocarpus siliculosus*, and diverse protists also produce siRNAs and miRNAs that potentially regulate molecular processes in the cell [17], [21], [22], [23], [24]. Therefore, the extent to which protists in general, and in particular algae already have a full breadth of regulatory systems based on RNAi remains an open question. To address this issue, we carried out a genome-wide analysis of sRNAs in the glaucophyte alga *Cyanophora paradoxa*. Because glaucophytes putatively occupy a basal phylogenetic position in the supergroup Plantae (also known as Archaeplastida) the RNAi system in this lineage may reflect ancestral and/or divergent features of the RNAi system described in green algae and plants [25], [26]. Therefore *C. paradoxa* offers an important model to study the evolution of sRNA-guided silencing in Plantae and more generally to understand the complexity of RNAi pathways in protists.

## Materials and Methods

### Growth Conditions

*Cyanophora paradoxa* (CCMP329) was cultivated at the Provasoli-Guillard National Center for Marine Algae and Microbiota (formerly CCMP). Using normal, replete growth conditions, cells were propagated under continuous light in standard DY-V medium to high density. Cells were then harvested by centrifugation and frozen in liquid nitrogen. To apply a high light shock, aliquots of high-density cultures were further exposed to daylight irradiance for 40 minutes before being frozen. Others aliquots were taken after 300 mM salt (NaCl) treatment for 30 minutes, and exposure to –2°C temperature for 40 minutes. These shock conditions represent strong environmental stimuli during relatively brief time intervals to ensure that differential gene expression was effectively elicited by the stresses.

### Library Preparation and Sequencing

The *TRIzol* method (Life Technologies, Grand Island, NY USA) was used to isolate total RNA from the replete-grown cells and from cultures submitted to light-, salt-, and cold-shock. RNA extracted from each treatment condition was fractionated by electrophoresis in 12% denaturing (7 M urea) polyacrylamide gel. Excision of a gel band in the range of 15–35 nt was used to select the fraction containing the cellular sRNAs of *C. paradoxa*. The *miRCat Small RNA Cloning Kit* (IDT, Coralville, IA, USA) was used to prepare sRNA libraries for each treatment condition according to the manufacturer’s instructions. A further PCR amplification step added indexed-*Illumina* (San Diego, CA, USA) adaptors necessary for sequencing on the GAIIx instrument. The mRNAs were purified from total RNA using *Dynabeads* (Life Technologies, Grand Island, NY USA). Degradome libraries for each treatment condition were prepared by ligation of a RNA linker to the 5′ phosphate of cleaved mRNAs [27]. The sequence of this linker (5′-ACACTCTTTCCCTACACGACGCTCTTCCGATCTNNNN-3′) corresponded to the *Illumina* Adapter 1 containing a customized index to identify the library. Reverse transcription was performed using an oligo containing a poly-d(T) fused to the Adapter 2 of *Illumina* (5′-CTCGGCATTCCTGCTGAACCGCTCTTCCGATCTTTTTTTTTTTTTTTTTTTTTTTT-3′). An enrichment step was performed using standard *Illumina* PCR primers. For preparation of RNA-seq libraries, cDNA was first synthesized from mRNAs of normal- and salt-treated cultures using the *Mint cDNA* synthesis kit (Evrogen, Moscow, Russia), and then converted to RNA-seq libraries by transposon tagmentation using the *Nextera* (Illumina, San Diego, CA, USA) DNA sample preparation kit. All libraries were sequenced on the *Illumina* GAIIx platform according to the manufacturer’s instructions.

### Bioinformatics

A bioinformatics pipeline for filtering sRNAs was established to demultiplex the sequenced sRNA libraries, trim the adaptor and barcodes, select sRNAs sequences of size 16–32 nt, and eliminate sRNAs that mapped to databases of rRNA and tRNAs and the plastid genome of *C. paradoxa*. Sequences of sRNAs and degradome tags were deposited at NCBI under the BioProject accession number PRJNA12793. Using the *CLC Genomics Workbench 5.5* (CLC bio, Aarhus, Denmark) the filtered sRNAs were mapped against the genome contigs, EST contigs, and predicted protein-coding sequences (CDSs) of *C. paradoxa*. Only full-length matches with 100% identity were considered for downstream analysis and gene expression studies. These sequences represent therefore, nuclear-encoded sRNAs in *C. paradoxa*. Degradome libraries were sequenced using the 50 bp single-read chemistry of *Illumina*. The resulting degradome data were also filtered to eliminate adaptors, library indexes, and rRNA/plastome hits. In addition, poly-A tails were removed from the degradome data when necessary. The same criterion of 100% identity was used to align degradome tags to the genome data, ESTs, and CDSs using the mapping tools of the *CLC Genomics Workbench.* This bioinformatics platform was also used for several other analysis reported here, such as quantification of sRNAs and degradome expression and sRNA redundancy analysis. The miRNAs were identified using *miRDeep* [28], and sRNA targets in the CDS of *C. paradoxa* were predicted using *CleaveLand 3* [29].

## Results

### Discovery of sRNAs in *Cyanophora paradoxa*

**Figure 1:**
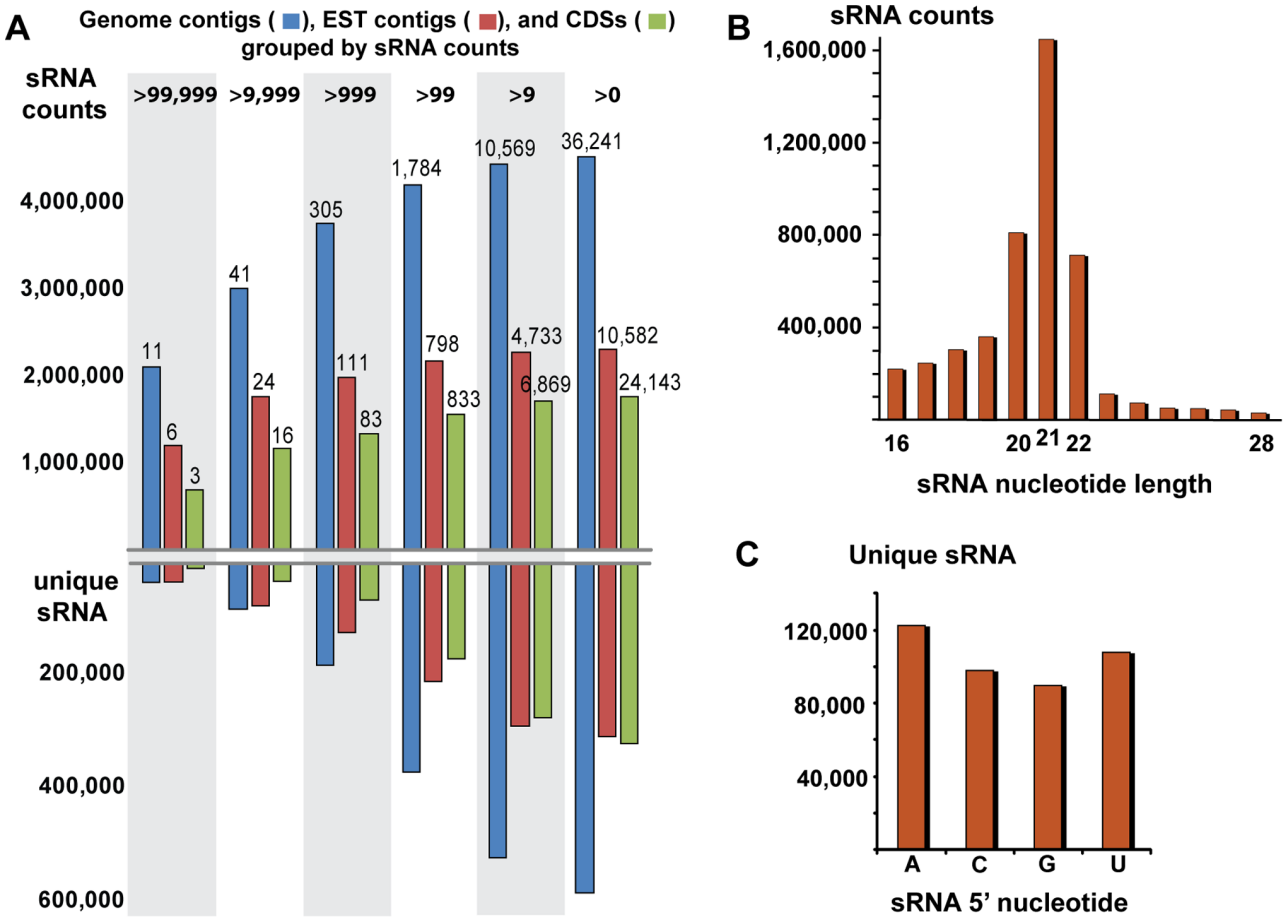
Mapping and composition features of sRNAs discovered in *Cyanophora paradoxa*. (A) The results of mapping redundant (above the x-axis) and unique (below the x-axis) sRNAs to genomic contigs, EST contigs, and CDSs. The numbers on the top of the colored bars correspond to the number of genomic contigs (blue), EST contigs (red) and CDSs (green) associated with the range of sRNAs counts displayed at the top of the panel. (B) Size distribution of redundant sRNAs in *C. paradoxa* indicates the predominance of the 21nt length class. (C) Composition of the 5′ nucleotide of unique sRNAs.

We recently produced a draft genome of *C. paradoxa* [25]. Homology searches using BLAST [30] against the assembled genome contigs of *C. paradoxa* reveal the presence of molecular components putatively involved in RNAi. When used as a query, the *A. thaliana* Dicer protein (AtDCL1, accession number NP_171612) retrieves a significant BLASTP hit (*e*-value 4×10^−9^) with a gene model predicted within the genome contig 53704, whereas sequences homologous to Argonaut (AtAGO1, AAC18440) are found on contigs 10646 and 53267, respectively. Surprisingly, the genome of *C. paradoxa* also encodes several putative homologs of *A. thaliana* RNA-dependent RNA polymerase (AtRDR1, NP_172932) located on genome contigs 42, 27852, 39851, and 9284. The presence in the *C. paradoxa* genome of genes homologous to key molecular components involved in RNAi provides strong evidence of an analogous system operating in this glaucophyte alga [17]. We sought therefore to characterize sRNAs from *C. paradoxa*. To accomplish this aim, four cDNA libraries were prepared from size-selected sRNAs extracted from cold-, light-, and salt-stressed algal cultures, and from cells grown under normal, replete conditions. These libraries were sequenced using the *Illumina* GAIIx platform. Upon filtering a total of 4,739,151 reads matched against genome data, EST contigs, and the predicted CDS from *C. paradoxa* (Table 1 and Figure 1A). Mapped sRNAs had the predominant size of 21 nt (Figure 1B) and a slight overrepresentation of adenine and uracil in the first base (Figure 1C). The widespread abundance of sRNAs and common characteristics suggest that *C. paradoxa* sRNAs have features also present in plants [11].

**Table 1:** The distribution of 4,739,151 sRNAs that were mapped to genome contigs, CDS sequences, and EST contigs.

Libraries	Mapped to 60,119 genome contigs		
	Redundant	Unique	Contigs mapped
Normal	1,696,901	121,147	14,704
Cold	287,434	93,924	15,407
Salt	1,246,244	299,703	30,565
Light	1,279,786	199,655	18,707
Total	4,510,365	584,976	36,241
	**Mapped to 31,895 CDSs**		
	**Redundant**	**Unique**	**CDSs mapped**
Normal	845,384	62,727	9,081
Cold	100,461	47,494	10,409
Salt	409,316	161,005	20,890
Light	407,496	100,456	12,916
Total	1,762,657	314,399	24,143
	**Mapped 15,003 EST contigs (>199 bp in length)**		
	**Redundant**	**Unique**	**ESTs mapped**
Normal	1,001,210	58,952	5,523
Cold	116,588	38,342	5,730
Salt	561,687	98,136	8,922
Light	623,155	97,314	7,844
Total	2,302,640	225,734	10,582

### Transcriptome-wide Mapping Reveals a Pathway for Exonic sRNA Production

**Figure 2:**
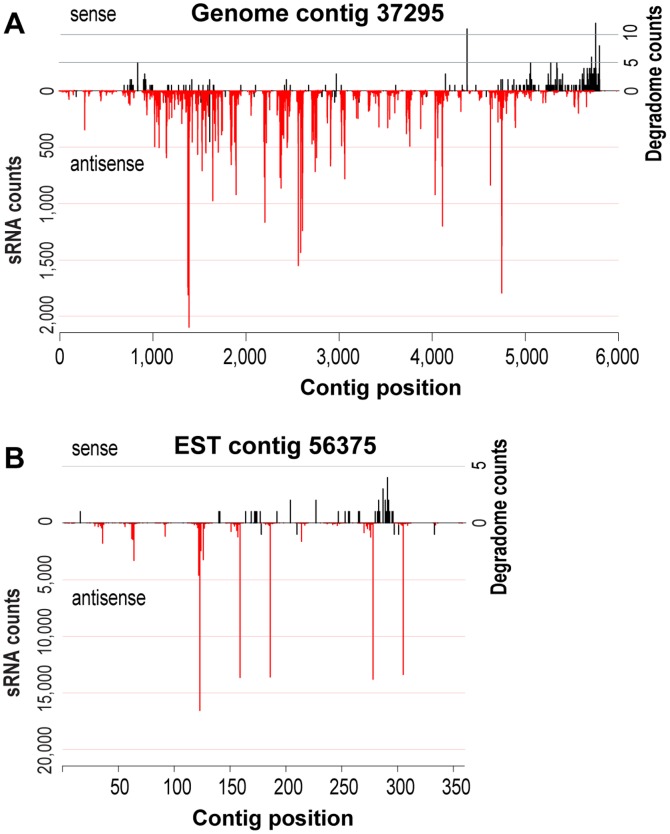
Two examples of sRNA and degradome mapping to *C. paradoxa* genome data. (A) Mapping of sRNAs (red) and degradome tags (black) to genome contig 37295. (B) Mapping of sRNAs (red) and degradome tags (black) to EST contig 56375.

**Figure 3:**
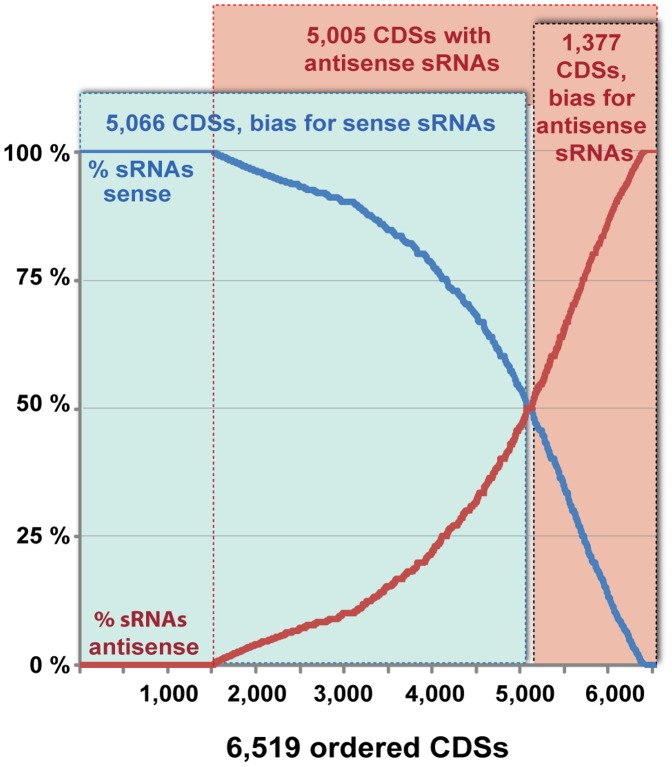
The sRNAs associated with CDSs show orientation bias. The percent of sRNAs that mapped in the sense (blue curve) or antisense (red curve) direction of 6,519 CDS selected on the basis of prevalent (>75%) degradome sense transcription or absence of antisense degradome transcription.

We mapped the four sRNA libraries to a data set of genome contigs, predicted CDSs, and EST contigs with size greater than 199 bp (Table 1, Figure 1A, and Table S1). More than one-half of the genome contigs (36,241 contigs) have at least one sRNA mapped to them. Surprisingly, a large fraction of the sRNAs is associated with a few regions of the genome. For instance, 3,749,680 redundant sRNAs map to only 305 of the total number of 60,119 genome contigs (Figure 1A). These 305 contigs, which encompass only 2.3% of the sequenced genome, are therefore associated with 83.13% of the total sRNAs, and likely represent hotspots for sRNA production. The mapping also reveals that sRNA production is primarily associated with transcribed regions. A total of 2,302,640 sRNAs (approximately 48.5% of the total) were mapped to over 70.0% of the selected EST contigs (Figure 1A, red bar in the middle). In addition, about 75.0% of the predicted CDSs have sRNAs associated with them, indicating that a large fraction of the sRNAs derive from exonic regions (Figure 1A, Table S1). In many instances, sRNAs that are associated with transcripts are biased to a specific DNA strand (e.g., genome contig 37295 and EST contig 56375 shown in Figures 2A and 2B, respectively). The correct transcriptional orientation can be determined using the degradome-seq data (Table S2) because the libraries were 5′ to 3′ directionally cloned [27]. In addition, the orientation of transcription is apparent using the predicted CDS. Based on these approaches, we found that many of the ESTs and CDSs have sRNAs mapped in an antisense orientation, ruling out the possibility that strand bias of sRNAs represents degradation of highly expressed mRNAs (Figure 3). Consistent with this idea, available mRNA-seq data from *C. paradoxa* indicate that some exonic sRNAs are associated with CDSs that have no expression data (Figure S1). Conversely, genes with high expression and degradation turnover do not necessarily give rise to sRNAs. Taken together these observations support the idea that a pathway for sRNA production from exons is present in *C. paradoxa*.

Notably, sRNAs in *C. paradoxa* are associated with protein-coding genes (Table S1). To get a better picture of this unusual pattern of sRNA distribution, we selected 6,519 CDSs for which degradome tags corroborate the orientation of the protein-coding gene (Figure 3). All these 6,519 CDSs have ≥10 sRNAs, many CDSs are associated with a large number of mapped sRNAs (the top 16 CDS in Table S1 have more than 10,000 mapped sRNAs). We found that 1,377 CDSs display a bias for antisense sRNA production (Figure 3). At least 107 CDSs have only antisense sRNA associated with them and over 900 CDSs have >20 antisense sRNAs. The top CDSs with the most sRNAs mapped to them show a strong predominance of antisense mapping (Table S1). In contrast, there are over 5,000 CDSs in which sRNAs are predominantly mapped in the sense orientation (Figure 3). About 3,500 of these CDSs also have antisense associated sRNAs and therefore may represent actual sites for sRNA production rather than a product of mRNA degradation.

This transcriptome-wide pattern of exonic sRNA with biased orientation remains unaffected when mapping unique (non-redundant) sRNAs to the 6,519 CDS (Figure S2), confirming that the production of sRNAs tends to spread across the transcript. We feel it is unlikely that these results are an artifact of cDNA over-amplification of a few sRNAs that map to particular sites of the CDS. Production of sRNAs across the length of an mRNA is consistent with the action of RdRPs [11], [12], four of which are potentially encoded in the genome of *C. paradoxa*. Although in some instances, we observed a pattern of blocks of mapped sRNAs over separate regions of the mRNA (e.g., EST contig 56375; Figure 2B), we did not detect a phasing distribution that is a typical signature of secondary siRNAs in plants [11], [12]. The pattern of exonic sRNAs seems to be a general trend affecting a large fraction of cellular mRNAs. For example, predicted genes with associated sRNAs encode proteins of diverse cellular functions including translation, signal transduction, and varied metabolic and cell biological functions (Table S1). Interestingly, the top CDSs with abundant sRNA associated with them do not have BLAST hits at NCBI therefore their identity and function are unknown (Table S1). This is also the case for many of the remaining sRNA-associated CDSs.

### Degradome Analysis Indicates the Predominance of a 5′ to 3′ mRNA Turnover Pathway

**Figure 4:**
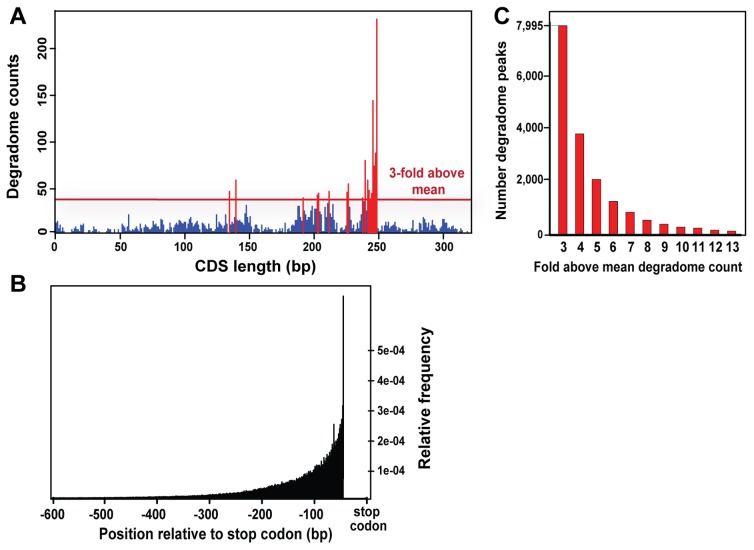
Profile of the *C. paradoxa* degradome tags mapped to predicted CDSs. (A) Example of degradome peaks distributed over CDS 7658.3. The blue peaks are tag counts <3x of the mean total number of peaks. The red peaks are tag counts ≥3x the mean total number of peaks. (B) Average contribution of total degradome peaks as a function of relative distance from the stop codon of CDSs. For this analysis, all individual CDSs tag counts for each position were numbered from the stop codon and normalized as a relative frequency to the total tag counts in that CDS. Then the total relative frequency for each position (relative to the stop codon) was calculated as an average number; i.e., the sum of individual relative frequencies in that position of all CDSs divided by the total number of CDSs. (C) For each *C. paradoxa* CDS, peaks with relative frequency of degradome tags ≥3x the mean total number of counts were retrieved (e.g., the red peaks for CDS 7658.6 on panel A). This resulted in 7,995 peaks from all CDSs (first bar on the left). The high-frequency degradome peaks are displayed as a function of the factor (3x–13x) by which their relative frequency surmounts the mean tag count found on the respective CDSs.

To gain insights into possible sRNA mediation of gene expression, we generated 5,166,650 degradome tags that were mapped to the genome contigs, CDSs, and EST contigs of *C. paradoxa* (Table S2). Of these, 836,255 degradome tags (from 25–45 nucleotides in length) had perfect matches to the predicted CDSs, thereby allowing for identification of reading-frame orientation. These tags were also tentatively used for the identification of possible sites of sRNA-mediated cleavage [29]. For this purpose, the degradome dataset was remapped to determine the 5′-phosphorylated position of the tag captured by our cloning procedure. This allowed us to generate for each CDS a graphic plot of peaks that represents the putative sites of mRNA cleavage (e.g., CDS 7658.3 in Figure 4A). The tendency for degradome peaks to be concentrated at 3′ ends of transcripts was revealed by plotting the relative frequency as a function of the distance to the stop codon of CDSs (Figure 4B). This bias in the distribution of degradome peaks is also found in *A. thaliana* and likely indicates overrepresentation of 5′ to 3′ exonucleolytic mRNA turnover products [27], [31].

**Figure 5:**
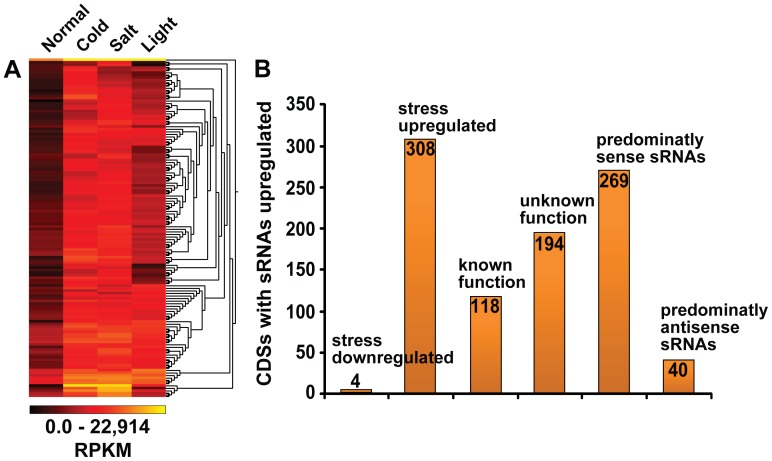
Expression analysis of sRNAs associated with CDSs. (A) Heat map showing results of cluster analysis of 312 filtered CDSs with differential expression of sRNAs. The filtering criteria were at least 100 normalized (reads per kilobase per million, RPKM) counts in one of the conditions and a difference of 10x fold or more of expression in at least one stress condition when compared to the normal. (B) Bars indicating how many differentially expressed CDSs are up-regulated or down-regulated under normal versus stress conditions, how many have known/unknown function, and the number of CDSs that have predominantly sense or antisense associated sRNAs.

In plants, the peaks of the degradome frequency distribution are usually significantly higher than the background level of exonucleolytic degradation [31], [32]. Therefore, to capture in *C. paradoxa* the potential signal from degradome peaks that may represent sRNA-mediated endonucleolytic products, we selected all peaks that were above background frequencies for each CDS. This was done by removing peaks that were below three-fold of the mean frequency of normalized counts per CDS. This procedure resulted in 7,995 peaks for downstream analysis (Figure 4C). We used a BLAST alignment approach to search for possible significant overlaps between sRNAs and the 7,995 high-frequency peaks. As a negative control, we generated 7,995 random peaks that were aligned to sRNAs using the same BLAST procedure. This analysis did not show a significant difference between the high-frequency group and the randomized dataset with respect to the number of sRNAs overlapping the degradome tags (results not shown). This analysis indicates that no obvious correlation exists between sRNAs and degradome peaks in *C. paradoxa*. To investigate the possibility that a few individual sRNAs may be associated with degradome peaks, we analyzed 100 highly expressed sRNAs in greater detail. We also used the *miRDeep* program [28] to identify 32 putative miRNAs in the genome of *C. paradoxa*. By running a targeting prediction under *CleaveLand 3* [29] we did not detect significant matches between our data set of 100 highly expressed sRNAs and 32 miRNAs to degradome tags when mapped to the CDSs. Our inability to find sRNA targets using different bioinformatic approaches suggests the possibility that in *C. paradoxa* sRNAs do not mediate mRNA cleavage. However, by analyzing expression profiles, we observed that exonic sRNAs related to CDSs appear to be differentially expressed between normal and stress conditions (i.e., salt, cold, and high light; Figure 5). To ensure that our analysis reflects meaningful differences in gene expression we constrained the analyzed dataset to CDSs that have 100 or more normalized sRNA counts in at least one of the four libraries (Figure 5). We found that 312 CDSs have at least 10-fold sRNA expression differences between the normal and one of the stress libraries, suggesting that the regulation of sRNA expression is influenced by environmental factors.

## Discussion

Here we describe genome-wide analysis of sRNAs in the glaucophyte alga *C. paradoxa*. The sRNAs we isolated mapped to over 70% of the EST contigs and to 75% of the predicted CDSs, demonstrating that sRNA production is associated with mRNA sequences in this species. The following lines of evidence indicate that the identified sRNAs are authentic exonic sRNAs rather than degradation products of abundantly expressed mRNA captured by our library construction procedure: (i) we found a global pattern of antisense sRNA biogenesis (Figure 3, Table S1). In many cases, individual CDSs were associated with thousands of sRNAs that have a predominant antisense orientation (Table 1). (ii) The mRNA-seq data indicate that some highly expressed genes do not produce a detectable sRNA (Figure S1). (iii) Conversely, some poorly expressed genes are the templates for significant sRNA production (Figure S1).

A possible explanation for the significant levels of transcript-derived sRNAs is the production of secondary siRNA by the action of RdRP during amplificatory cascades of the RNAi signal [11], [12]. BLAST analysis indicates that *C. paradoxa* putatively encodes 4 loci with sequence identity to *A. thaliana* RdRP homologs, suggesting that transitivity may exist in this alga. The phenomenon of secondary siRNA production from RNAs that were targeted by a miRNA or primary siRNA is very important in plants to efficiently suppress viral infection in the cell [1], [11]. In addition, in plants RdRP production of secondary siRNA has been co-opted for regulatory networks involving a few protein-encoding genes in the cell. In *A. thaliana* there are 5 TAS *loci* that are targeted by specific miRNAs, triggering recruitment of RdRP and the production of multiple secondary siRNAs [11], [12]. These tasiRNAs target and regulate the expression of genes involved in the auxin system and modulate developmental processes [11], [33]. TasiRNAs also target pentatricopeptide repeat (PPR) protein encoding transcripts [34]. Many of these PPR mRNAs show prolific production of secondary siRNAs that are functionally dependent on the catalysis of RdRPs. Despite the overrepresentation of sRNAs among the PPR family, only about 20% of protein-coding genes in *A. thaliana* have sRNAs associated with them [35]. From this pool, approximately 75% of the genes (i.e., 15% of all protein encoding genes) have ≤5 sRNA counts. These numbers suggest that the association of sRNAs with protein-coding transcripts is modest even among multicellular taxa such as *A. thaliana* that encodes 6 RdRP homologs. However exonic sRNAs are abundant in the fungus *Mucor circinelloides* where they are produced by RdRPs, mediating the silencing of targeted mRNAs [10], [36]. The lack of genetic tools in *C. paradoxa* precludes identification of the molecular circuitry involved in the production of exonic sRNAs to advance our understanding of the role of these molecules in regulating algal gene expression. However our finding that sRNAs are predominantly associated with mRNAs and the identification of putative RdRPs in *C. paradoxa* suggests that a mechanism must exist for the production of secondary siRNAs. If this is confirmed then we speculate that transitivity represents an ancestral trait present in the common ancestor of Plantae. The likely presence of transitivity in *C. paradoxa* and in *M. circinelloides* also suggests that a complex RNAi system was present in the ancestor of all eukaryotes.

## Supporting Information

Figure S1 The frequency of sRNA counts and gene expression levels are not correlated. (A) From RNA-seq analysis (data not shown) we observed 4,700 CDSs that do not have gene expression counts while displaying different levels of sRNA expression (shown on the x-axis). (B) In contrast, there are 3,867 CDSs that have RNA-seq counts (i.e. they are expressed) but do not result in sRNAs. Note that in 16 cases, highly expressed genes (>1,000 RNA-seq counts) do not produce any sRNAs.
(PDF)

Figure S2 The percentages of strand prevalence of both redundant and unique sRNAs to transcriptionally oriented CDSs and ESTs. (A) For the sake of comparison, a similar plotting to Figure 3 is shown. The 6,519 CDS were selected on the basis of prevalent (>75%) degradome sense transcription or absence of antisense degradome transcription. (B) From the previous 6,519 CDSs, 4,720 CDSs were selected that produced 10 or more unique sRNAs (non-redundant). The same trend of skewed strand distribution of sRNAs is still maintained and therefore is not an artifact of overamplification of redundant sRNAs species. (C) Mapping done against EST contigs (>199 bp length). The 2,995 ESTs that have 70% or more degradome tags that determine the transcriptional direction were ordered on the x-axis. The percent of sense/antisense sRNA indicates the strand specificity of many sRNAs. (D) The same scenario of strand bias is maintained when choosing 2,465 ESTs that have 10 or more sRNAs associated with them.
(PDF)

Table S1 Transcriptome-wide mapping of exonic sRNAs in *C. paradoxa*.
(XLSX)

Table S2 Degradome mapping to genomic contigs, EST contigs >199 bp in length, and predicted CDSs from *C. paradoxa*.
(DOCX)
