# Supplementary material for: Evidence for Widespread Exonic Small RNAs in the Glaucophyte Alga Cyanophora paradoxa
Source: PLoS One. 2013 Jul 3;8(7):e67669. doi: 10.1371/journal.pone.0067669 (PMC3700990; doi:10.1371/journal.pone.0067669)
Supplement: Figure S1 — The frequency of sRNA counts and gene expression levels are not correlated. (A) From RNA-seq analysis (data not shown) we observed 4,700 CDSs that do not have gene expression counts while displaying different levels of sRNA expression (shown on the x-axis). (B) In contrast, there are 3,867 CDSs that have RNA-seq counts (i.e. they are expressed) but do not result in sRNAs. Note that in 16 cases, highly expressed genes (>1,000 RNA-seq counts) do not produce any sRNAs. (PDF) [file pone.0067669.s001.pdf]

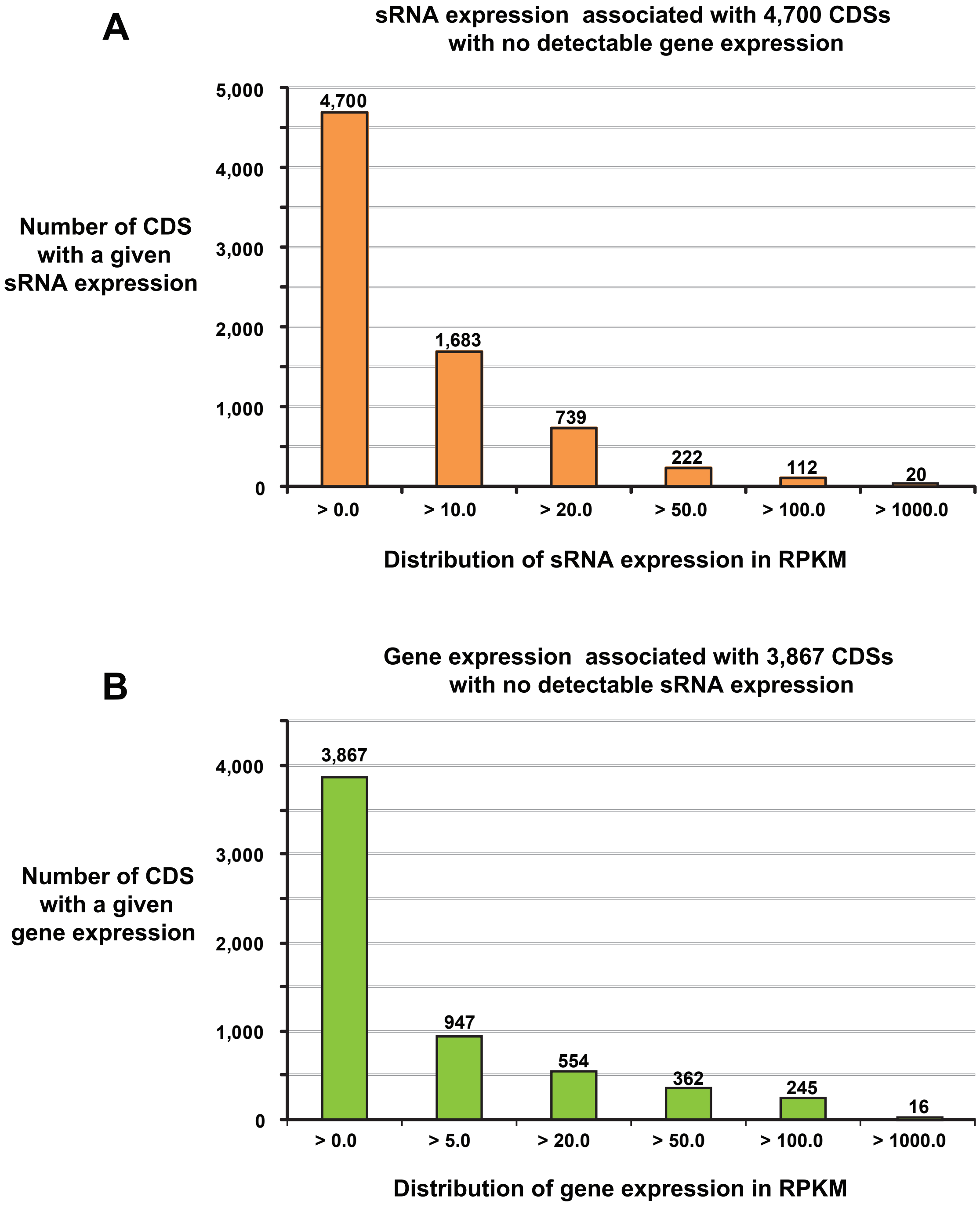

Figure S1. The frequency of sRNA counts and gene expression levels are not correlated. (A) From RNA-seq analysis (data not shown) we observed 4,700 CDSs that do not have gene expression counts while displaying different levels of sRNA expression (shown on the x-axis). (B) In contrast, there are 3,867 CDSs that have RNA-seq counts (i.e. they are expressed) but do not result in sRNAs. Note that in 16 cases, highly expressed genes (> 1,000 RNA-seq counts) do not produce any sRNAs.
