# Supplementary material for: Evidence for Widespread Exonic Small RNAs in the Glaucophyte Alga Cyanophora paradoxa
Source: PLoS One. 2013 Jul 3;8(7):e67669. doi: 10.1371/journal.pone.0067669 (PMC3700990; doi:10.1371/journal.pone.0067669)
Supplement: Figure S2 — The percentages of strand prevalence of both redundant and unique sRNAs to transcriptionally oriented CDSs and ESTs. (A) For the sake of comparison, a similar plotting to Figure 3 is shown. The 6,519 CDS were selected on the basis of prevalent (>75%) degradome sense transcription or absence of antisense degradome transcription. (B) From the previous 6,519 CDSs, 4,720 CDSs were selected that produced 10 or more unique sRNAs (non-redundant). The same trend of skewed strand distribution of sRNAs is still maintained and therefore is not an artifact of overamplification of redundant sRNAs species. (C) Mapping done against EST contigs (>199 bp length). The 2,995 ESTs that have 70% or more degradome tags that determine the transcriptional direction were ordered on the x-axis. The percent of sense/antisense sRNA indicates the strand specificity of many sRNAs. (D) The same scenario of strand bias is maintained when choosing 2,465 ESTs that have 10 or more sRNAs associated with them. (PDF) [file pone.0067669.s002.pdf]

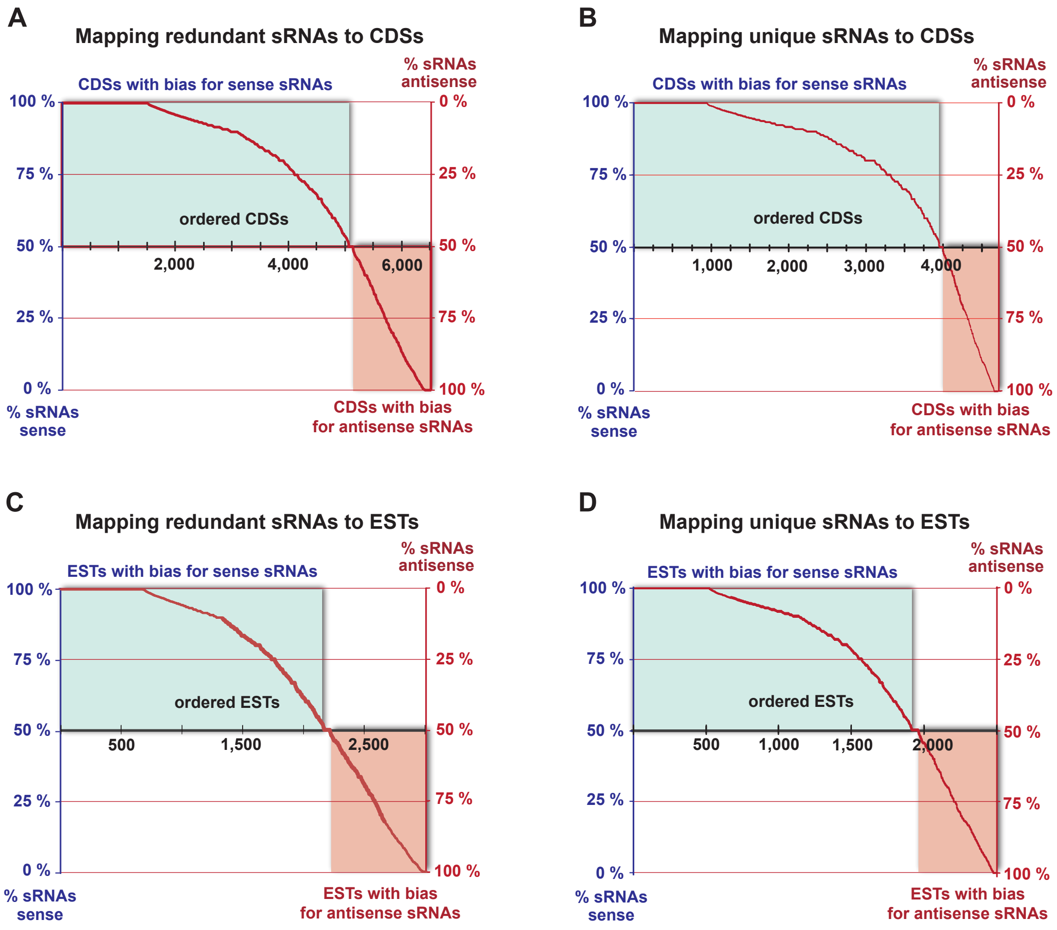

Figure S2. The percentages of strand prevalence of both redundant and unique sRNAs to transcriptionally oriented CDSs and ESTs.

(A) For the sake of comparison, a similar plotting is used as shown in Figure 3. The 6,519 CDS were selected on the basis of prevalent (>75%) degradome sense transcription or absence of antisense degradome transcription. (B) From the previous 6,519 CDSs, 4,720 CDSs were selected that produced 10 or more unique sRNAs (non-redundant). The same trend of skewed strand distribution of sRNAs is still maintained and therefore is not an artifact of overamplification of redundant sRNAs species. (C) Mapping done against EST contigs (>199 bp length). The 2,995 ESTs that have 70% or more degradome tags setting the transcriptional direction were ordered on the x-axis. The percent of sense/antisense sRNA indicates the strand specificity of many sRNAs. (D) the same scenario of strand bias is maintained when choosing 2,465 ESTs that have 10 or more sRNA counts associated with them.
