# Supplementary material for: Evidence for Widespread Exonic Small RNAs in the Glaucophyte Alga Cyanophora paradoxa
Source: PLoS One. 2013 Jul 3;8(7):e67669. doi: 10.1371/journal.pone.0067669 (PMC3700990; doi:10.1371/journal.pone.0067669)
Supplement: Table S2 — Degradome mapping to genomic contigs, EST contigs >199 bp in length, and predicted CDSs from C. paradoxa. (DOCX) [file pone.0067669.s004.docx]

| **Table S2.** Degradome mapping to genomic contigs, EST contigs >199 bp in length, and predicted CDSs from *C. paradoxa*. | | | | | | |
| --- | --- | --- | --- | --- | --- | --- |
|  | **Mapped to 60,119 genomic contigs** | | **Mapped to 31,895 CDSs** | | **Mapped 15,003 EST contigs (>199 bp)** | |
| **Libraries** | **Degradome tags** | **Contigs mapped** | **Degradome tags** | **CDSs mapped** | **Degradome tags** | **ESTs mapped** |
| **Normal** | 1,184,503 | 12,602 | 180,880 | 5,244 | 529,422 | 7,619 |
| **Cold** | 1,582,816 | 11,752 | 173,104 | 5,486 | 1,245,774 | 7,525 |
| **Salt** | 193,157 | 10,267 | 46,167 | 4,084 | 97,429 | 6,493 |
| **Light** | 1,817,363 | 13,672 | 436,056 | 6,267 | 1,023,906 | 8,220 |
| **Total** | 4,777,839 | 15,904 | 836,255 | 7,871 | 2,896,435 | 9,027 |
